# Supplementary material for: Psychosocial Distress in Adult Patients Awaiting Cancer Surgery during the COVID-19 Pandemic
Source: Curr Oncol. 2021 May 13;28(3):1867–78. doi: 10.3390/curroncol28030173 (PMC8161781; doi:10.3390/curroncol28030173)
Supplement: Supplementary file 1 [file curroncol-28-00173-s001.zip › curroncol-1191310-supplementary.pdf]

Supplemental Material

Semi-structured Interview Guide.

1. Before we get started, I wonder if you can tell me what is happening with you during this time? Family life, work life, changes that have happened in your life due to COVID?

2. Tell me more about your understanding of your diagnosis

3. Tell me about your experience since you heard your surgery was delayed because of the COVID-19 pandemic.
   a. How were you told?
   b. What was your initial reaction?

4. Did you have concerns at that time about your surgery being delayed?
   a. IF YES: Tell me more about your concerns.
   How are you feeling now about your surgery being delayed?

5. What has been most difficult for you during this time?

6. How would you describe your emotional well-being right now?
   Worry, stress
   Fear of progression (tumor growing or cancer getting worse)
   Frustration, confusion, anger
   Loneliness
   Anxiety
   Depression

7. Has your situation led to changes in your physical health or behaviours?
   Sleep disturbance
   Irritability
   Appetite changes
   Headaches

8. Have you spoken to your surgeon or health care team about your concerns or your well-being?
   a. IF YES: What did they say?
   b. IF NO: Why not?

9. How are you managing during this time?
   Speaking with cancer care team
   Speaking with your primary care provider
   Spouse/partner/people in your household
   Video chatting with family/friends
   Prayer
   Physical activity / exercise / yoga
   Mindfulness / meditation
   Eating healthy
   Maintaining routine
   Hobbies

Any other ways

10. Thinking about when you get your surgery date – what do you think about going to the hospital around the time of COVID?
    Concerned?
    What are you concerned about?

11. Would you consider changes in treatment or your care approach because of these delays, if your surgeon were to bring alternatives up? For example, undergoing ongoing monitoring to see if you cancer increases in size rather than going to surgery right away?
    If that were possible, what would you think about it?

12. Is there anything else about your experience right now, specifically around your surgery being delayed, that we haven't discussed but you would like to tell me about?

Reflexivity Statements.

Sarah Murnaghan

As the research assistant, my primary role in this study was conducting the interviews. My background is in nursing (BScN), though not in the area of oncology. I am a Registered Nurse employed by Nova Scotia Health. My gender is female. I am a novice researcher, and student in the MSc program within the Department of Community Health and Epidemiology at Dalhousie University, where Dr. Urquhart is my thesis supervisor. Training and guidance on qualitative research were given by Dr. Urquhart. There was no relationship between me and any participant prior to study commencement. Personal goals for participating in the research were to gain experience in qualitative methodology and practice in the research environment. There are no further characteristics to report about myself, other than those stated.

David Forner

As the first author, my role in this study was that of study design, data analysis, and manuscript preparation. I am a white male in my 30's who was raised in Eastern Canada. My training background is in biology and chemistry (BSc Hon), medicine (MD), and clinical epidemiology (MSc). I am an otolaryngology – head & neck surgery resident physician and work with patients every day in a clinical capacity. Some of these patients have similar diagnoses to participants in the study. Furthermore, I have a strong interest in shared decision-making and related research, as well as in head and neck cancer, both clinically and in a research capacity. I have a strong desire to improve the lives of my current and future patients, and to identify areas in research which may assist in this goal. There were no relationships between myself and any of the participants before the study, and I have never acted as their physician.

Robin Urquhart

I am an experienced cancer health services researcher, and hold the Canadian Cancer Society Endowed Chair in Population Cancer Research. My gender is female. I have >15 years of experience in qualitative and mixed methods research, and mentored Dr. David Forner and Sarah Murnaghan on this study. I have worked with a number of co-authors on other cancer-related research projects. There was no relationship between me and any study participant.

Table S1. Supporting codes used throughout the qualitative analysis with accompanying domains.

Domain	Codes
**Communication and trust with/of health care team**	Communication with surgeon / healthcare team (pertaining to surgery)<br>Communication with medical oncologist<br>Communication with primary care provider<br>Informed of COVID delay (or not)<br>Reassurance from surgeon / healthcare team<br>Trust (or lack-of) in surgeon
**COVID challenges**	Social isolation<br>Disruption to everyday tasks<br>Navigating the healthcare system<br>Inability to continue with normal routine
**Patient factors**	Patient understanding (of their diagnosis, prognosis, risk status, …)<br>Familiarity (e.g., cancer in family, had procedure before, etc.)<br>Patient expectations (e.g., knowing what to expect from hearing things in the media, etc.)<br>Wanting to "get it done" (i.e., surgery)<br>Cancer history<br>Fear of surgery (or not wanting it)<br>Comorbidities (social factors or additional health problems) (e.g., diabetes, single mother, on well-fare, etc.)
**Patient health and well-being**	Emotional health & well-being<br>• Level of COVID-related worry / anxiety (e.g., economy, state of province or world).<br>• Fear of progression<br>• Frustration<br>• Other emotional issues (e.g., after surgery, loss of kidney)<br>Physical health and well-being<br>Lack of control, unknown or uncertainty
**Coping mechanisms & supports**	Family or friend support<br>Spirituality or faith<br>Keeping busy, keeping routines<br>Activity, exercise<br>Other coping mechanisms / supports
**Hospital factors during COVID**	Hospital restrictions or prioritization of resources<br>Risk of catching COVID
